# Prediction and validation of potential molecular targets for the combination of *Astragalus membranaceus* and *Angelica sinensis* in the treatment of atherosclerosis based on network pharmacology

**DOI:** 10.1097/MD.0000000000029762

**Published:** 2022-06-30

**Authors:** Tianyue Wang, Yaqiong Zhou, Kaina Wang, Xinyu Jiang, Jingbo Wang, Jing Chen

**Affiliations:** a The 2nd Clinical Medical College, Zhejiang Chinese Medical University, Hangzhou, China; b Pan’an People’s Hospita, Jinghua, China; c The 1st Clinical Medical College, Zhejiang Chinese Medical University, Hangzhou, China; d Library, Zhejiang Chinese Medical University, Hangzhou, China; e School of life science, Zhejiang Chinese Medical University, Hangzhou, China.

**Keywords:** *Angelica sinensis*, *Astragalus membranaceus*, atherosclerosis, network pharmacology, molecular docking

## Abstract

Since the 20^th^ century, mortality rate due to cardiovascular diseases has increased, posing a substantial economic burden on society. Atherosclerosis is a common cardiovascular disease that requires urgent and careful attention. This study was conducted to predict and validate the potential molecular targets and pathways of *Astragalus membranaceus* and *Angelica sinensis* (A&A) in the treatment of atherosclerosis using network pharmacology. The active ingredients of A&A were obtained using the TCMSP database, while the target genes of atherosclerosis were acquired using 2 databases, namely GeneCards and DrugBank. The disease-target-component model map and the core network were obtained using Cytoscape 3.8.2 and MCODE plug-in, respectively. The core network was then imported into the STRING database to obtain the protein-protein interaction (PPI) network diagram. Moreover, gene ontology (GO) and Kyoto encyclopedia of genes and genomes (KEGG) enrichment analyses were performed using the HIPLOT online website. Finally, the small molecules related to key signaling pathways were molecularly docked and visualized. Under the screening conditions of oral bioavailability ≥ 30% and drug-likeness ≥ 0.18, 22 active ingredients were identified from A&A, and 174 relevant targets were obtained. Additionally, 54 active ingredients were found in the extracted core network. Interleukin (IL)-17 signaling pathway, tumor necrosis factor (TNF) signaling pathway, and Toll-like receptor (TLR) signaling pathway were selected as the main subjects through KEGG enrichment analysis. Core targets (RELA, IKBKB, CHUK, and MMP3) and active ingredients (kaempferol, quercetin, and isorhamnetin) were selected and validated using molecular docking. This study identified multiple molecular targets and pathways for A&A in the treatment of atherosclerosis. A&A has the potential to treat atherosclerosis through an antiinflammatory approach.

## 1. Introduction

Cardiovascular diseases have been the leading cause of death worldwide since the beginning of the 20^th^ century, accounting for more than twice the number of cancer-related deaths and imposing a huge economic burden on society and the healthcare sector.^[1]^ Atherosclerosis is a common cardiovascular disease.^[2]^ Because atherosclerosis is a chronic inflammatory disease with a complex and interrelated mechanism, its pathophysiology is not fully understood. The most striking feature of atherosclerosis is plaque formation, where inflammation, oxidative stress, and autoimmune functions are contributing factors.^[3]^ Plaque formation in atherosclerosis undergoes the following 4 stages: endothelial cell dysfunction, lipoprotein deposition and oxidation, inflammatory factor effects, and fibrous cap development.^[4]^ Atherosclerosis mainly occurs in the coronary arteries. The continuous growth of plaques and the instability of rupture may cause local blood flow destabilization, angina, myocardial infarction, and even cardiac arrest that can endanger the life of patients.^[5]^ Currently used pharmacological treatments target the inflammatory response to atherosclerosis and control the excessive stress of immune cells to protect the blood vessels.^[6]^

Several drugs are available to treat this disease, but the primary treatment option is statin-based lipid-lowering drugs that can counter the inflammatory response in atherosclerosis.^[6]^ Chinese herbs have been widely used in clinical practice in Asian countries, especially China. However, due to a lack of rigorous clinical trials and unclear pharmacological mechanisms, many western countries still have reservations about Chinese medicine. For traditional Chinese medicine (TCM), combination therapy using different herbs may offer new treatment options for atherosclerosis. In TCM, the treatment methods for atherosclerosis rely on activating blood, replenishing qi, clearing heat, and removing stasis,^[7–9]^ which are proven to have specific curative effects. Compared with a single drug, TCM combines several drugs and the small molecules in different herbs, which may act synergistically to facilitate the treatment of diseases.

The combination of A&A is widely used in TCM prescriptions for treating cardiovascular diseases. The main ingredients of *Astragalus membranaceus* (AM) include polysaccharides, saponins, and flavonoids, which have antiinflammatory and antioxidant effects.^[10]^ AM exhibits significant treatment effects in colitis, diabetes, and kidney disease and protects the cardiovascular and respiratory systems.^[11–13]^ The present study shows that AM can interfere with AMP-activated protein kinase (AMPK), nuclear factor-κB (NF-кB), and other signaling pathways to reduce inflammation and excessive immune response.^[14]^ Another traditional Chinese herb, *Angelica sinensis* (AS), has long been used to tonify and invigorate the blood, relieve pain, and moisten the intestines.^[15]^ AS extracts have been shown to protect the liver,^[16]^ treat cerebral ischemia-reperfusion injury,^[17]^ reduce inflammation, and alleviate atherosclerosis.^[18,19]^

**Figure 1.**
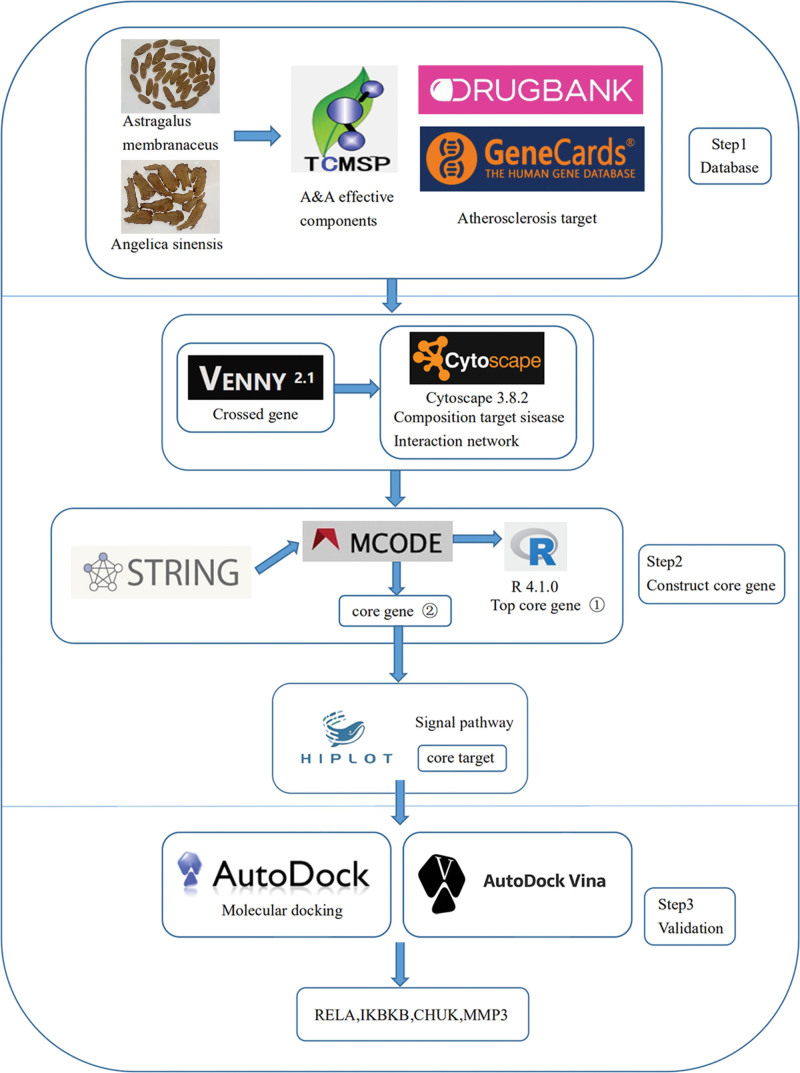
Network pharmacology analysis workflow.

TCM uses a combination of A&A for treating atherosclerosis. However, the underlying mechanism is not yet elucidated due to the lack of relevant studies. Network pharmacology is an analytical method that combines a biological network of systems with pharmacology, and it reflects the holistic philosophy of TCM.^[20,21]^ In this study, network pharmacology was used to elucidate the potential mechanism of the combination of A&A in the treatment of atherosclerosis and reveal the potential targets and pathways. The detailed workflow diagram of the study is presented in Figure 1.

## 2. Materials and Methods

### 2.1. Establishment of a database for A&A target genes and atherosclerosis-related genes

The TCMSP database^[22]^ (https://tcmspw.com/tcmsp.php) was applied to select 2 drugs, *Astragalus* and *Angelica*, by setting oral bioavailability (OB) ≥ 30% and drug-likeness (DL) ≥ 0.18. Genes related to atherosclerosis were collected by using DrugBank (https://www.drugbank.ca/) and GeneCards databases (https://www.genecards.org/MyGenes/) with the keyword “Atherosclerosis.”

### 2.2. Network construction of Chinese herbal medicines and their components

The components of each herbal medicine obtained using the TCMSP database were imported into Cytoscape 3.8.2 software, and the ingredients present in each drug were visualized and analyzed.

### 2.3. Construction of common targets between A&A and atherosclerosis

The UniProt database (https://www.uniprot.org/) was used to transform the names of the collected disease variant genes and drug targets and to make them compatible. The genes were then imported into the “VENNY 2.1” online tool (https://bioinfogp.cnb.csic.es/tools/venny/index.html) to obtain the common genes.

### 2.4. Construction of a component-target-disease interaction network of A&A with atherosclerosis

The previously acquired common genes and the associated active ingredients were visualized and analyzed using Cytoscape 3.8.2 software.

### 2.5. Construction of protein-protein interaction (PPI) network graphs

The prior collection of common genes was entered into the STRING online database (https://string-db.org/), and the species was selected as “*Homo sapiens*.” The results were then imported into Cytoscape 3.8.2 software in “tsv” format to analyze the core network further.

### 2.6. Construction of a core network

The data collected from previous steps were sorted in order from the most to least number of nodes, and the top 20 were picked to create a histogram using “*R*” 4.1.0. The “tsv” file was imported into Cytoscape 3.8.2 software, and the MCODE plug-in was applied to analyze the core network. The top-ranked network was selected as the core network for the next step.

### 2.7. GO and KEGG pathway enrichment analyses

The core genes were imported into the HIPLOT online analysis website (https://hiplot.com.cn), where the KEGG database was selected, and the species was set to *Homo sapiens*. The results reflected the biological processes, molecular functions, cellular compositions, and pathways implicated during the treatment of atherosclerosis. The results are presented as a bubble or bar chart; the smaller the *P*-value, the higher the enrichment; the larger the bubble, the richer the genes are.

### 2.8. Molecular docking

The study of active ingredients of A&A and their targets for the treatment of atherosclerosis using molecular docking techniques can help illustrate the mechanism of action and binding activity of active ingredients and target proteins to a certain extent.^[23]^ According to IL-17, TNF, and TLR signaling pathways, the pathways obtained from KEGG enrichment were selected. Their targets and the corresponding active molecules were collected. The structures of the compounds (kaempferol, quercetin, isorhamnetin, formononetin, beta-sitosterol, 7-O-methylisomucronulatol, and Calycosin) were downloaded from the PubChem database (https://pubchem.ncbi.nlm.nih.gov/) in SDF format and then converted to mol2 format using Chem 3D software. The structures of the proteins (RELA, IKBKB, CHUK, MMP3, JUN, MAPK14, FOS, AKT1, TNF, CASP3, IL1B, MMP9, IL6, CASP8, CXCL8, CCL2, and CXCL10) were downloaded from the RCSB database (https://www.rcsb.org/) in PDB format. Pymol software was applied to remove solvent molecules and ligands, and AutoDock Tools 1.5.6 software was used to add hydrogen, calculate the charge, and assign atomic types. Finally, the results were saved in pdbqt format. Autodock vina 1.1.2 was used for molecular docking, and the 6 combinations with the lowest binding energy were selected for visualization and analysis of the docked conformations using Discovery Studio 2020.

## 3. Results

### 3.1. Active ingredients of A&A and the herbal medicine network

**Figure 2.**
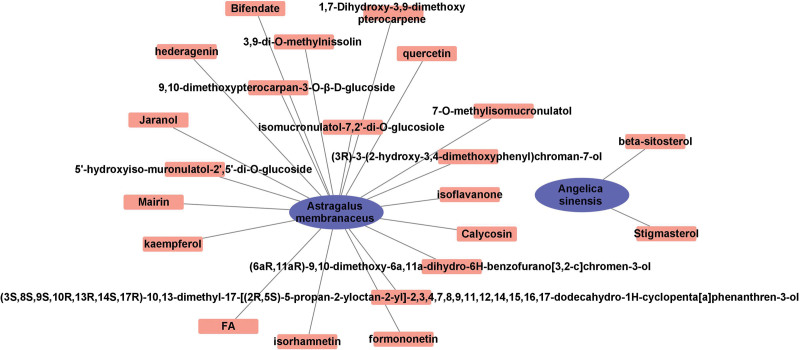
Active ingredients in A&A and the Chinese herbal medicine network.

AM and AS were searched separately in the TCMSP database to screen for active ingredients meeting an OB value ≥ 30% and a DL ≥ 0.18. The results showed 20 active ingredients in AM (mairin, jaranol, hederagenin, (3S,8S,9S,10R,13R,14S,17R)-10,13-dimethyl-17-[(2R,5S)-5-propan-2-yloctan-2-yl]-2,3,4,7,8,9,11,12,14,15,16,17-dodecahydro-1H-cyclopenta[a]phenanthren-3-ol, isorhamnetin, 3,9-di-O-methylnissolin, 5’-hydroxyiso-muronulatol-2’,5’-di-O-glucoside, 7-O-methylisomucronulatol, 9,10-dimethoxypterocarpan-3-O-β-D-glucoside, (6aR,11aR)-9,10-dimethoxy-6a,11a-dihydro-6H-benzofurano[3,2-c]chromen-3-ol, bifendate, formononetin, isoflavanone, calycosin, kaempferol, FA, (3R)-3-(2-hydroxy-3,4-dimethoxyphenyl)chroman-7-ol, isomucronulatol-7,2’-di-O-glucosiole, 1,7-dihydroxy-3,9-dimethoxy pterocarpene, and quercetin), and 2 active ingredients in AS (beta-sitosterol and stigmasterol). The network diagram is shown in Figure 2.

### 3.2. Cross-targets of A&A and atherosclerosis

**Figure 3.**
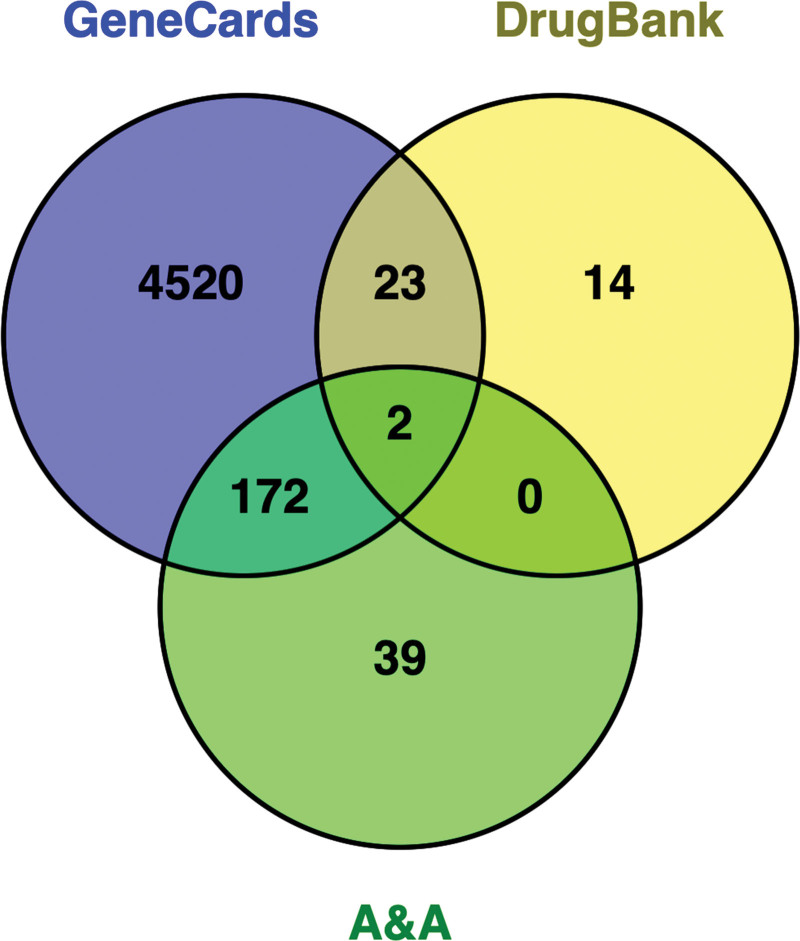
Venn diagram for the cross-validation targets of A&A and atherosclerosis.

The eligible active ingredients were retrieved from the TCMSP database, and the targets corresponding to the relevant active components were collected in this database. Finally, 213 targets were obtained. When “Atherosclerosis” was used as the keyword, 4717 and 39 targets were obtained using the GeneCards and DrugBank databases, respectively. The above targets were entered into the online site of venny 2.1 to obtain the common targets, as shown in Figure 3. It was clear that A&A had 172 and 2 common targets within GeneCards and DrugBank, respectively. In summary, 174 targets related to A&A were identified as primary targets for further research.

### 3.3. Network of interactions between A&A and atherosclerosis

**Figure 4.**
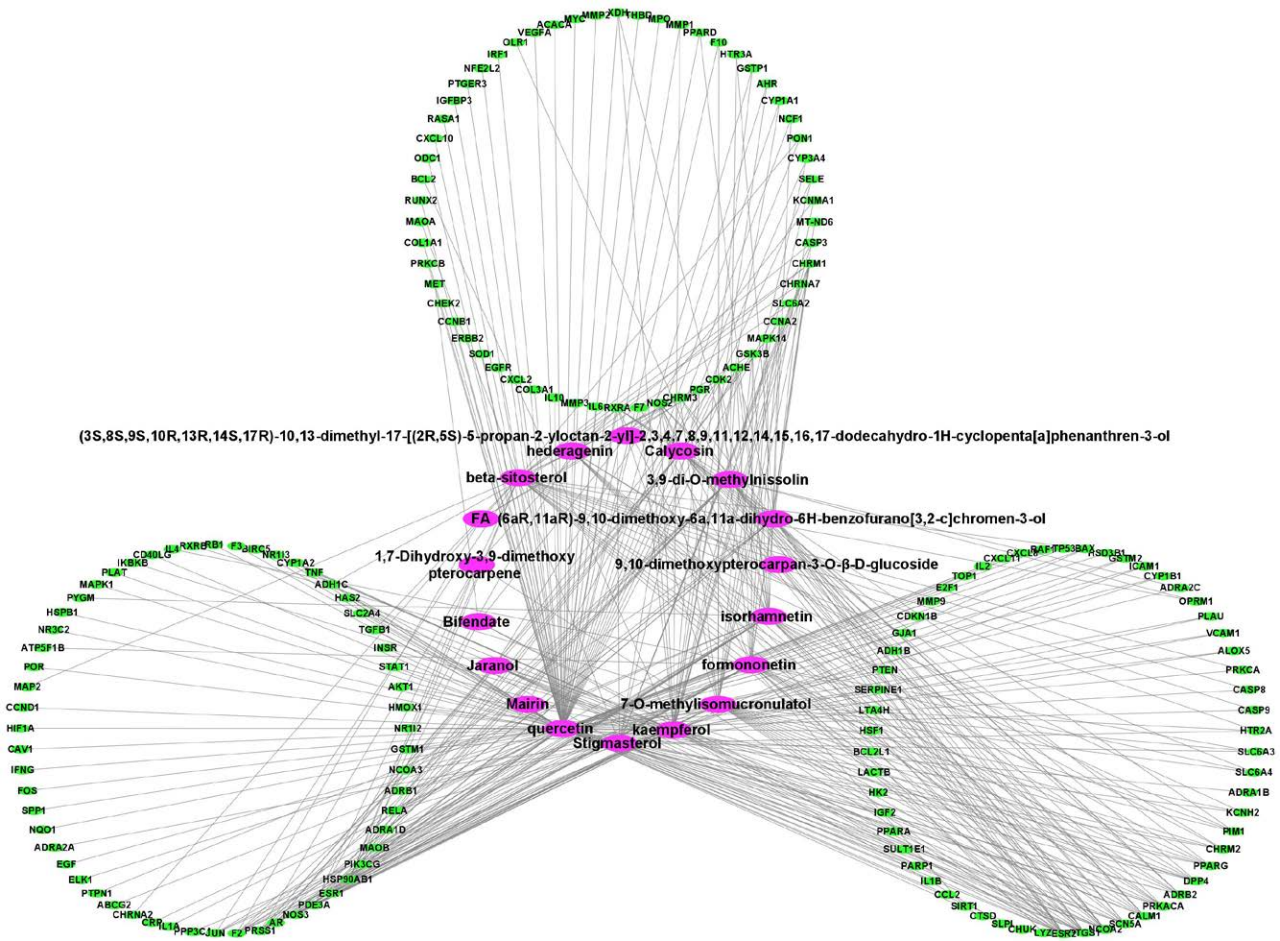
Ingredient-target-disease interaction network of A&A and atherosclerosis. The purple nodes represent the active ingredients and the green nodes represent the targets.

The 174 common targets identified for active ingredients from 18 drugs were imported into Cytoscape 3.8.2 software for visualization and analysis. As shown in Figure 4, the active ingredients in drugs were marked purple, and the targets were marked green. The drug-target network diagram clearly shows a link between the drug and the disease.

### 3.4. Core network

**Figure 5.**
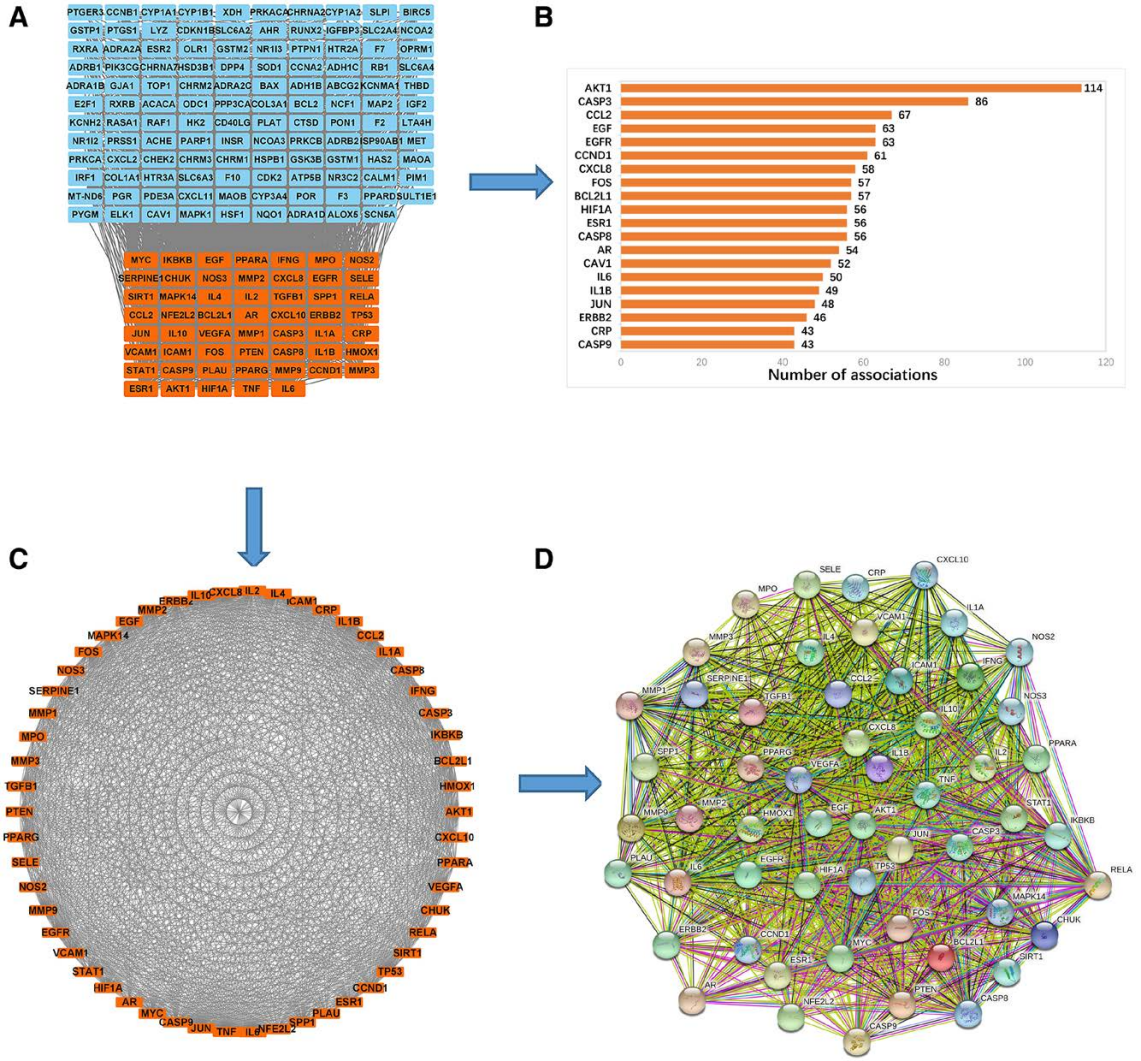
Analysis of core genes at the intersection of A&A regulatory genes and atherosclerosis target genes. (A) A&A regulatory genes and atherosclerosis target genes intersection. (B) Barplot statistical results of the interaction between A&A and atherosclerosis regulatory genes. (C) Core genes of A&A and atherosclerosis. (D) PPI network of core genes and corresponding proteins.

The 213 common genes collected through Venn diagram analysis were entered into the STRING online database, with the species selected as “*Homo sapiens*.” The data from the preliminary analysis were exported in “tsv” format for further study using Cytoscape 3.8.2, as shown in Figure 5A (1 gene does not have a target associated with it and is therefore not displayed). The orange color corresponds to the top-ranked core network analyzed by the MCODE plug-in. The final score for this core network was 43.132, with 54 nodes and 1143 edges. The “tsv” file was processed using *R* 4.1.0, and the top 20 proteins with the highest number of associations in this network were selected to plot the histogram, as shown in Figure 5B. After taking the core network out separately, their corresponding interactions are shown in Figure 5C.

### 3.5. PPI network analysis

The 54 proteins related to the core network obtained from the previous MCODE plug-in analysis were entered into the STRING database for PPI network analysis, and the results are shown in Figure 5D. The PPI network had an average node degree of 42.3, an average local clustering coefficient of 0.865, an expected edge count of 305, and a PPI enrichment *P*-value < 1.0 e-16. In the resulting graph, the darkness of the node depicts its importance in the network (i.e., the darker the node is, the more critical it is in the network).

### 3.6. GO analysis and KEGG pathway enrichment analysis

**Figure 6.**
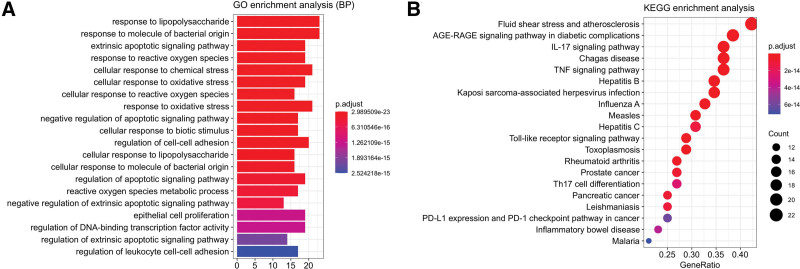
Enrichment analysis. (A) GO analysis of A&A and atherosclerosis target genes. (B) KEGG analysis of A&A and atherosclerosis target genes.

GO and KEGG analyses were performed using the HIPLOT online analysis website. The KEGG database (public/db/kegg/hsa_kegg_20210326.rds) with the species selected as “*Homo sapiens*” was used to analyze the relevant genes. GO analysis involves biological processes (BP), cell composition (CC), and molecular function (MF). The analysis results of the top 20 most significant relevant BP terms are presented as bar charts, as shown in Figure 6A. Similarly, KEGG selected the top 20 pathways to be represented as bubble plots, as shown in Figure 6B.

### 3.7. Validation of the interaction of active ingredients with target genes

**Figure 7.**
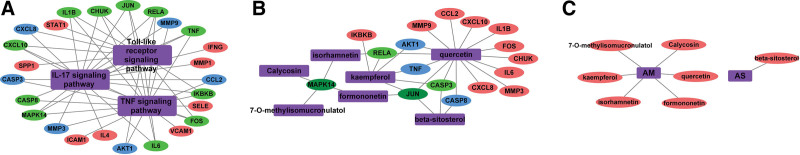
Correlation analysis of core signaling pathways, targets, and active ingredients. (A) Key signaling pathways and target genes of A&A and atherosclerosis intersection genes. (B) Active ingredients corresponding to key targets. (C) Association of key active ingredients with A&A.

The enrichment results for the signaling pathways were selected to be further analyzed for 3 significant pathways, IL-17, TNF, and TLR signaling pathways. The genes related to these 3 pathways were imported into Cytoscape 3.8.2 software for visualization and analysis. Figure 7A shows 25 genes associated with the 3 pathways. The associations between the 7 relevant active ingredients in the drug and the 25 genes were identified, as shown in Figure 7B. There were 6 and 1 components associated with AM and AS, respectively, as shown in Figure 7C.

**Figure 8.**
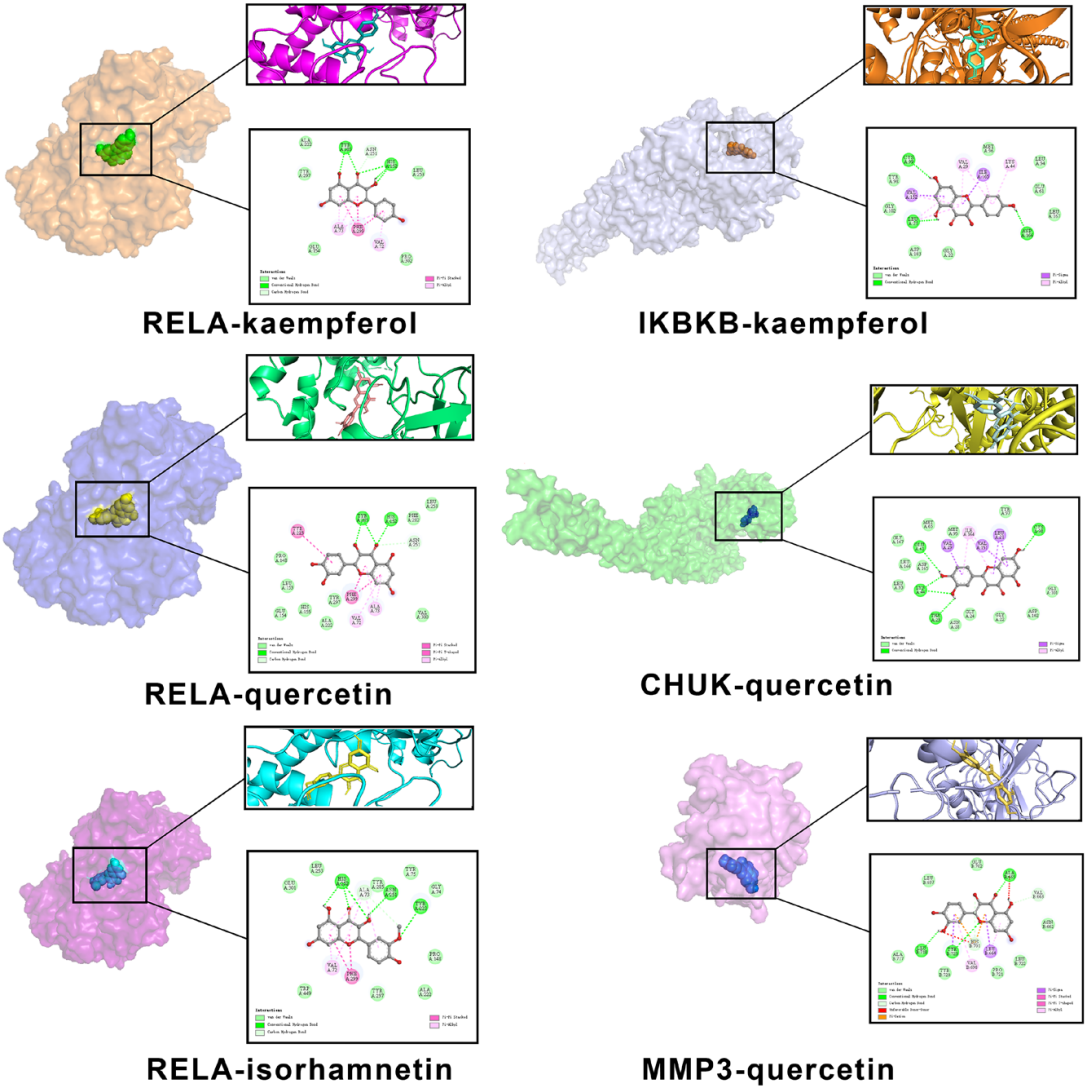
Molecular docking of key target genes and ingredients: The proteins RELA and IKBKB interacted with kaempferol. The proteins RELA, CHUK, and MMP3 interacted with quercetin, and the protein RELA interacted with isorhamnetin.

The 7 active ingredients were docked to their relevant target proteins using molecular docking techniques. The docking results are shown in Table 1, where lower binding energy indicates a more stable binding. The docking combinations were ranked from the least to most in terms of binding energy, and the top 6 docking combinations were selected for visual analysis. The results are shown in Figure 8.

**Table 1:** The combination of the best docking model energy.

Compounds	Target proteins	PDB ID	Binding energy (kcal/mol)
Kaempferol	RELA	3QXY	–9.4
Kaempferol	IKBKB	4KIK	–9.3
Quercetin	RELA	3QXY	–9.2
Quercetin	CHUK	5EBZ	–9
Quercetin	MMP3	1HY7	–9
Isorhamnetin	RELA	3QXY	–9
Formononetin	JUN	2G01	–8.9
Kaempferol	JUN	2G01	–8.6
Isorhamnetin	MAPK14	3DT1	–8.6
Quercetin	FOS	5PAM	–8.4
Quercetin	JUN	2G01	–8.4
Beta-sitosterol	JUN	2G01	–8.4
Quercetin	AKT1	4GV1	–8.3
Formononetin	MAPK14	3DT1	–8.1
Kaempferol	AKT1	4GV1	–7.8
7-O-methylisomucronulatol	MAPK14	3DT1	–7.8
Calycosin	MAPK14	3DT1	–7.8
Kaempferol	TNF	2AZ5	–7.7
Quercetin	TNF	2AZ5	–7.7
Kaempferol	CASP3	4PS0	–7.4
Quercetin	CASP3	4PS0	–7.4
Beta-sitosterol	CASP3	4PS0	–7.4
Quercetin	IL1B	5R85	–7.2
Quercetin	MMP9	4JIJ	–7.2
Quercetin	IL6	1N26	–7
Quercetin	CASP8	4PS1	–6.7
Quercetin	CXCL8	6WZM	–6.2
Beta-sitosterol	CASP8	4PS1	–5.9
Quercetin	CCL2	1DOK	–5.9
Quercetin	CXCL10	1O80	–5.9

In the combination of CHUK-quercetin, quercetin formed hydrophobic interactions with Ile164, Pi-Sigma interactions with Val29, Val151, and Leu21, and hydrogen bonds with Glu61, Lys44, Thr23, and Cys98 of CHUK. This combination exhibited the best binding effect among the 6 groups, with a binding energy of -9.4 kcal/mol. During IKBKB-kaempferol docking, kaempferol formed hydrophobic interactions with Val152, Leu21, Val29, Ile165, and Lys44, Pi-sigma interactions with Val152 and Ile165, and hydrogen bonding interactions with Cys99, Leu21, and Asp166 of IKBKB. On the other hand, quercetin connected to MMP3 by forming hydrophobic interactions with Val698 and Leu664, Pi-cation interactions with His701, Pi-sigma interactions with Tyr723 and Leu664, and hydrogen bonding interactions with Leu718, Tyr723, and Ala665 of MMP3. The RELA protein could link to most molecules, including isorhamnetin, kaempferol, and quercetin, all being stably docked. Isorhamnetin formed hydrophobic interactions with Ala73 and Val72 of RELA, hydrogen bonding interactions with His252, Asn251, Tyr223, and Ala73, and a Pi-Pi stacking effect with Phe299. Kaempferol formed hydrophobic interactions with Ala73 and Val72, hydrogen bonding interactions with His252, Asn251, Tyr223, and Ala73, and a Pi-Pi stacking effect with Phe299. RELA docking with quercetin showed that quercetin would form hydrophobic interactions with Val72 and Ala73, hydrogen bonding interactions with Tyr285, His252, and Asn251, a Pi-Pi T-shaped effect with Tyr223, and a Pi-Pi stacking effect with Phe299.

## 4. Discussion

TCM is a complex system encompassing small molecules with complex pharmacological activities (synergistic or antagonistic), their targets, and the pathways of action.^[8]^ Network pharmacology is widely used to study the mechanisms of action of Chinese medicine in treating diseases,^[24–26]^ by systematic elaboration of relevant targets and active ingredients, in line with the holistic philosophy of Chinese medicine. The initial network of interaction was obtained, followed by the computerized molecular docking techniques to simulate the docking of the most representative active ingredients with their associated targets. This step can verify the feasibility of the selected drugs for disease treatment.

This study was based on a network pharmacology approach to investigate the active ingredients and potential targets of A&A in the treatment of atherosclerosis. Twenty-two active ingredients were included in the screening criteria using TCMSP database analysis, of which 20 were from Astragalus and 2 were from Angelica. The DrugBank and GeneCards databases were used to identify 174 effective targets of A&A for treating atherosclerosis. For the analysis of the key mechanisms of action of the drug, the MCODE plug-in in STRING was used to extract the core network having the top score according to computer calculation, which can be seen in Figure 5C. The core network was used to construct the PPI network diagram (Fig. 5D). The enrichment of GO and KEGG was in the range of core genes, agreeing to the satisfactory result for the core network.

GO enriched and demonstrated the most critical 20 BP in this study. KEGG also enriched the related pathways. For further research, we selected 3 crucial signaling pathways, IL-17, TNF, and TLR signaling pathways. The development and progression of atherosclerosis are reported to be strongly related to the cellular factors and inflammatory response in the body, and the associated treatment can focus on controlling inflammation.^[27–30]^ IL-17 is the main cytokine produced by Th17 and tcrγδ + t cells. IL-17 inhibits fungal infections to a certain extent and exhibits pro-inflammatory properties.^[31,32]^ Therefore, high levels of IL-17 in the blood lead to heightened inflammatory responses, promoting plaque growth in the blood vessels or making the plaque unstable and aggravating atherosclerosis.^[33,34]^ TNF and TLR signaling pathways are associated with the NF-кB signaling pathway. The most crucial factor of the TNF signaling pathways is TNF-α. Upregulation of TNF-α can activate NF-кB,^[35–37]^ causing p65 in the NF-кB to enter the nucleus and trigger inflammatory gene expression.^[38,39]^ Similarly, TLR acts as a signal receiver and transmitter and contributes to the signal transduction and activation of the NF-кB signaling pathway in response to external inflammatory factors, resulting in an increased inflammatory response in the vasculature.^[40,41]^

The 3 signaling pathways mentioned above have essential research implications in the treatment of atherosclerosis. Therefore, we used molecular docking techniques to dock all the active components related to these 3 pathways and obtained the binding energy magnitude between the active small molecule substances and the target proteins, as detailed in Table 1. In general, binding energies below -5.0 kcal/mol demonstrated that the 2 molecules are well connected, and the values below -7.0 kcal/mol indicated that the binding activity of the 2 molecules is strong. From the docking results, the binding energies of CASP8-quercetin, CXCL8-quercetin, CASP8-beta-sitosterol, CCL2-quercetin, CXCL10-quercetin were higher than -7 kcal/mol and lower than -5 kcal/mol, while the rest showed strong activity. These results indicated that A&A has a strong potential to treat atherosclerosis through the IL-17, TNF, and TLR signaling pathways.

The present study predicted the active ingredients, targets, and signaling pathways involved in the treatment of atherosclerosis through network pharmacology. It also validated the feasibility of related small molecules that showed promising results in the molecular docking analysis. The feasibility and scientific validity of the combination of the 2 drugs (A&A) in the treatment of atherosclerosis were revealed. The core network and related targets highlighted in this study could serve as a guideline for other drugs used to treat atherosclerosis. However, our study also has some limitations. The accuracy of information in the database involved in collecting relevant data might have affected our conclusions. More experimental verification of the effects of the active ingredients in both drugs is also needed to obtain more reliable evidence for the efficacy of A&A in the treatment of atherosclerosis.

## 5. Conclusions

The treatment of atherosclerosis by A&A involved multiple compounds, targets, and pathways. The use of network pharmacology revealed the potential action mechanisms and targets for A&A in the treatment of atherosclerosis. This study also found that the positive effects of A&A in atherosclerosis are, at least in part, due to the antiinflammatory effects of this combination.

## Author contributions

Conceptualization: Tianyue Wang, Yaqiong Zhou, Jingbo Wang, Jing Chen

Data curation: Kaina Wang, Xinyu Jiang

Funding acquisition: Jing Chen

Methodology: Tianyue Wang, Yaqiong Zhou, Kaina Wang, Xinyu Jiang

Project administration: Jingbo Wang, Jing Chen

Software: Tianyue Wang, Yaqiong Zhou

Supervision: Kaina Wang, Xinyu Jiang

Visualization: Tianyue Wang, Yaqiong Zhou, Kaina Wang, Xinyu Jiang, Jingbo Wang, Jing Chen

Writing – original draft: Tianyue Wang, Yaqiong Zhou

Writing – review & editing: Tianyue Wang, Yaqiong Zhou

## Acknowledgment

The authors are indebted to the editor and reviewers for their constructive comments. We also thank TopEdit (www.topeditsci.com) for its linguistic assistance during the preparation of this manuscript.
